# Letter from M. Vernois

**Published:** 1840-07-04

**Authors:** 


### FOREIGN CORRESPONDENCE.

### LETTER FROM M. VERNOIS.

No. III.—[*Concluded.*]

*The Late Concours for the Chair of Internal Pathology in the School of Medicine of Paris.*

Paris, 27th April, 1840.

To the Editors of the Medical Examiner.

In the first part of this letter, we gave an account of the results of the two first trials of the candidates for the chair of internal pathology in the School of Medicine of Paris, which, as we mentioned, consisted first in the composition of a written essay on *Fever*, and the delivery of a lecture after a day's preparation. The third trial demanded an extemporaneous effort of the candidates—namely, a lecture *ex abrupto*, after three hours' reflection. This trial has an important end in view, despite of the obloquy cast upon it by many. It is true that it is, in some measure, a repetition of that which precedes it; but it affords the judges an opportunity of appreciating the solidity of a candidate's acquisitions. At a musical concours, the competitors would be expected to play every description of music at sight, and no less demand should be made of a professor of medicine. No medical subject should be unfamiliar to him; and this trial is the touchstone of the practical value of his acquirements. As, in this trial, the time is much more limited, so ought the questions to be of less scope; and they were shaped with considerable tact in this respect. In general, each candidate had a *symptom* to discuss. The following were the questions: hæmoptysis; strictures and occlusions of the intestines; menorrhagia; asthma; the urine in diseases; gangrene. The results of this trial were several capital lectures. It gives us great pleasure to specify those of M. Guillot upon diseased urine; M. Gendrin on intestinal strictures; M. Dalmas upon asthma. The rest were much inferior, displaying little erudition or felicity of arrangement.

The severest test to which the candidates were subjected, was the *thesis*. We shall give a rapid analysis of the results. M. Guillot had for a subject *the influence of pathological anatomy upon therapeutics*. This thesis was well written, and logically sustained. The author gave an excellent definition of pathological anatomy, taking evidently the view which should at this day be taken of it. The study of the alterations, of which the solids, liquids, and gases which make up the body are susceptible, was the basis of his essay; and he afterwards attempted to show how, and under what relations, the knowledge of the greater part of, if not all, these lesions, had successively modified therapeutics. M. Dalmas had to discuss the subject of *metastases*. This very obscure topic of pathology was extremely well elucidated in the essay of M. Dalmas. Placed on the one hand between old ideas, and on the other the progressive extension which the subject has received in our day, it was M. Dalmas' task to arrange and classify known facts, to attempt to give them an interpretation more precise and general, and thus subject them to an explanation more rational, and more in keeping with the progress of medical science. He commenced by defining metastases. According to him, they exist, whenever, with the cessation more or less rapid, of a physiological and pathological phenomenon, there coincides the appearance of new disorders in some other part of the body. Thence he proceeded to examine metastases in particular. *Order 1st.*—The menstrual, urinary, biliary, milky, perspiratory, gaseous. *Order 2d.*—The hæmorrhagic, purulent, congestive, inflammatory, nervous. Finally, in twenty-six general propositions, the author makes a rapid and lucid resumé of all his points. M. Legroux treated the subject of the nature of specific diseases. The candidate began by remarking that he had not had assigned to him as a subject, specific diseases, but their general character,—a distinction of practical importance. Under the head assigned him, he attempted to comprise the law which governs not only the affections ordinarily termed specific, but also those in which there supervenes, either in the symptoms or progress, something so peculiar, that this fact alone suffices to give it a *special*, and hence a truly *specific* character. This is not, as must be seen, to confound the terms specific and special, but to show their analogy and relationship with each other. M. Legroux made the following distinctions in his subject.

1st. A specific character, absolute and constant in causes and diseases; 2d, that which is relative, (special, properly so called,) resulting from accidental modifications in the characters of ordinary diseases. And here the specific is no longer the distinctive character of the species, but of the variety in the species. After the elucidation of these principles, M. Legroux gives the characters of the specific in diseases, and studies all the circumstances by the aid of which we are enabled to determine its development and appearance. M. Requier had to treat of the premonitory symptoms *in* diseases. We think that the author erred in the course he pursued. Taking the terms of his subject *literally*, he has admitted the following divisions: 1st, of premonitory phenomena; 2dly, premonitory symptoms; 3dly, premonitory diseases. These doctrines were maintained with talent by the candidate, but they certainly militate with sound medical language and fundamental principles. On the periodical character of diseases, was assigned to M. Hourmann. His thesis presented nothing remarkable, but was a faithful exposé of what is contained on this subject in all the treatises on etiology. It was well written and arranged. M. Cazenave had to discuss revulsives and derivatives. This was rather a threadbare subject,—many special treatises have been published on it,—and, perhaps, the author, instead of attempting a new path, would have done better to confine himself to a résumé, able as it must have been, of the actual state of our knowledge on the topic. The desire to do better than this, involved him in a definition so extensive, that it comprises at least all the known remedies for disease. In fact, after having given the same acceptation to the terms derivation and revulsion, he says:—" These terms I shall consider as synonymous to signify a complex organic act, in which the physiological or normal condition of a part is diminished, modified, or altered, owing to a normal or abnormal organic cause, spontaneously supervening, or artificially produced, in some other part." After some pages on the history of revulsives, come the divisions under which are separately studied every species of revulsives: 1st, by pain; 2dly, by congestion; 3dly, by inflammation; 4thly, by modification of the circulation; 5thly, by increase of organic action; 6thly, by particular organic action. Finally, M. Cazenave attempted to give the laws of revulsion, and terminated by the exposition of a theory of the action proper of this remedy. M. Piorry had for a subject the hereditary nature of diseases. This important, but almost endless subject, was not understood by the candidate. The following were his divisions. In the first place, he attempted to determine what is to be understood by the hereditary character in diseases, and those diseases themselves. He studied the circumstances which may favour their development and simulate their existence, and terminated by a theory on the subject. In the second section, he settled what are the diseases which exercise a general influence upon the economy; then passed to the study of the affections, the particular seat of which is more or less established. Then he spoke of the hereditary character in diseases, that is to say, considered in its relations with their various phenomena. Finally, he devoted some pages to the therapeutic means proper to control the hereditary aptitude to a disease, prevent its development, or cure it when it has appeared. M. Broussais treated of statistics as applied to pathology and therapeutics. His thesis, the materials of which were unfortunately unfit for analysis, was one of the most remarkable essays that we have read. M. C. Broussais declares himself the partisan of statistics, which he considers an indispensable instrument of the experimental method of observation in medicine. M. Gibert, who wrote upon the alterations of the blood, gave a rapid analysis of the works published on this subject, but his own production was far from complete. M. Dubois, of Amiens, had fluxes and congestions to treat of. His thesis was very voluminous, and almost exclusively devoted to the history of this chapter of science. The following are the principal points of view under which he considered the subject:—1st, historical and critical researches upon fluxes and congestions; 2dly, physiological and pathological history of fluxes and congestions; 3dly, anatomical and pathological history of congestions; 4thly, therapeutic indications, applicable to the treatment of these affections. This thesis must always be considered as a work of importance, and valuable for consultation. M. Gendrin, so successful in the previous trials, was not equally so in his thesis on the influence of age upon diseases. It was reduced to propositions, and unfit for analysis. M. Combette closed the list by a discussion on dropsy. This thesis was a resumé of the state of the science on the subject, but contained many errors and omissions.

In the trial, the account of which we have finished, the judges again showed their liberality. Every subject afforded scope for the development of an important question, and gave occasion to some capital theses. The discussion was, in general, lively and brilliant. MM. Dalmas, Guillot, Dubois of Amiens, Requier, and Broussais distinguished themselves in it.

A last test remained—that founded on an estimate of the anterior claims of each candidate. Among them were many men of prominence; we may mention MM. Gendrin, Piorry, Dalmas, C. Broussais, who, by their works and lectures, had acquired a well deserved reputation. Should, however, all other qualifications be sacrificed to this? Were the results of a trial, prolonged to four months, to be forgotten? We think not. Such, however, was the case. On Wednesday, the 26th of March, after a deliberation prolonged for four hours, the President of the concours announced the success of M. Piorry. The following were the results of the ballotings. On the first ballot, M. Casimer Broussais had 5 votes, M. Piorry 3, M. Dubois of Amiens, 4, M. Gibert, 1. On the second, Broussais, 2, Piorry, 5, Dubois, 5. On the third, Piorry, 6, Dubois, 6. The casting vote of the President, M. Duméril, decided the question in favour of M. Piorry.

This nomination has been, in general, badly received. In fact, whatever be the merits of the new professor, he possesses none of the brilliant and solid qualities requisite for distinction in the chair of pathology; and among the candidates there was ample room for a choice of greater promise, both of brilliancy and usefuluess.

